# The reverse-mode NCX1 activity inhibitor KB-R7943 promotes prostate cancer cell death by activating the JNK pathway and blocking autophagic flux

**DOI:** 10.18632/oncotarget.9806

**Published:** 2016-06-03

**Authors:** Zhou Long, BaiJun Chen, Qian Liu, Jiang Zhao, ZhenXing Yang, XingYou Dong, LiuBin Xia, ShengQuan Huang, XiaoYan Hu, Bo Song, LongKun Li

**Affiliations:** ^1^ Department of Urology, Second Affiliated Hospital, Third Military Medical University, Chongqing, 400037, China; ^2^ Department of Gastroenterology, First Affiliated Hospital, Medical College of Chengdu, Chengdu, 610500, China; ^3^ Department of Urology, First Affiliated Hospital, Third Military Medical University, Chongqing, 400038, China

**Keywords:** autophagy, apoptosis, prostate cancer, NCX1, cell death

## Abstract

We explored the effects of KB-R7943, an inhibitor of reverse-mode NCX1 activity, in prostate cancer (PCa). NCX1 was overexpressed in PCa tissues and cell lines, and higher NCX1 levels were associated higher PCa grades. At concentrations greater than 10 μM, KB-R7943 dose-dependently decreased PC3 and LNCaP cell viability. KB-R7943 also increased cell cycle G1/S phase arrest and induced apoptosis in PC3 cells. KB-R7943 increased autophagosome accumulation in PCa cells as indicated by increases in LC3-II levels and eGFP-LC3 puncta. Combined treatment with chloroquine (CQ) and KB-R7943 decreased P62 and increased LC3-II protein levels in PC3 cells, indicating that KB-R7943 blocked autophagic flux. KB-R7943 induced autophagosome accumulation mainly by downregulating the PI3K/AKT/m-TOR pathway and upregulating the JNK pathway. In xenograft experiments, KB-R7943 inhibited tumor growth. Combined treatment with KB-R7943 and an autophagy inhibitor inhibited growth and increased apoptosis. These results indicate that KB-R7943 promotes cell death in PCa by activating the JNK signaling pathway and blocking autophagic flux.

## INTRODUCTION

Prostate cancer (PCa) is among the most common causes of death in males worldwide, with the highest mortality rates occurring in developed countries [1, 2]. In recently years, PCa rates have increased dramatically in China [3]. Unfortunately, the pathogenesis of prostate cancer is not fully understood. Additionally, prostate cancer often is not diagnosed until late stages, contributing to poor prognoses [4, 5]. Uncovering the molecular mechanisms of PCa may lead to much-needed novel therapeutic approaches.

Autophagy is the process by which mammalian cells degrade and reuse old organelles. Autophagy dysfunctions are related to many diseases and affect cell survival, aging, and tumor and neurodegenerative disorders [6]. The role of autophagy in cancer is controversial. It has been reported that cancer cells use autophagy to survive nutrition shortages. Excessive autophagy not only promotes tumor growth, but also increases resistance to anti-cancer treatments [7, 8]. Therefore, inhibition of autophagic flux has been proposed as a novel therapeutic strategy for the treatment of cancer [9].

Intracellular free Ca^2+^ plays a central role in many fundamental cellular processes, including cell proliferation, differentiation, gene transcription, and cell death [10]. Therefore, disturbances of Ca^2+^ homeostasis mechanisms often lead to disorders, including cancer [11]. Indeed, the role of Ca^2+^ is well-established in many cell signaling pathways and in many types of cancer [12]. The Na^+^/Ca^2+^ exchanger (NCX) is a plasma membrane protein expressed ubiquitously in mammalian cells that maintains intracellular Ca^2+^ balance under different physiological conditions. The three different NCX isoforms (NCX1, NCX2 and NCX3) can either extrude Ca^2+^ from the cytosol in exchange for Na^+^ (forward-mode) or vice versa (Ca^2+^ in and Na^+^ out, reverse-mode) [13, 14]. However, NCX most often works in reverse-mode in pathological conditions, including arrhythmia, diabetes, and cancer [15–17]. As described these reports, most NCX research has focused on cardiovascular and nervous system diseases, and its role in cancer is poorly understood. However, recent studies suggested that reverse-mode NCX activity is prevalent in neuroblastoma cells and cardiac fibroblasts, and the resulting Ca^2+^ influx upregulated ERK1/2 activity [18, 19]. In addition, Ca^2+^ influx through cell membrane NCX is required for VEGF-induced ERK1/2 phosphorylation and PKCa-induced angiogenesis in endothelial cells. These studies reveal that NCX can promote tumor angiogenesis by activating ERK1/2 downstream of thrombin and angiopoietin [20]. Therefore, reverse-mode NCX activity plays an important role in cancer. NCX1 is overexpressed in some cancer cells and tissues, such as colorectal cancer and pancreatic cancer [21, 22], but the role of NCX1 in prostate cancer remains unknown.

In previous studies, we observed aberrant NCX1 expression in prostate cancer tissues. Additionally, KB-R7943, an inhibitor of reverse-mode NCX1 activity, played a major role in cardiac ischemia and injury [23]. Here, we examined whether KB-R7943 affected PCa development by altering reverse-mode NCX1 activity.

## RESULTS

### NCX1 expression is increased in prostate cancer tissues and KB-R7943 induces autophagosome accumulation in prostate cancer cells

**Figure 1:**
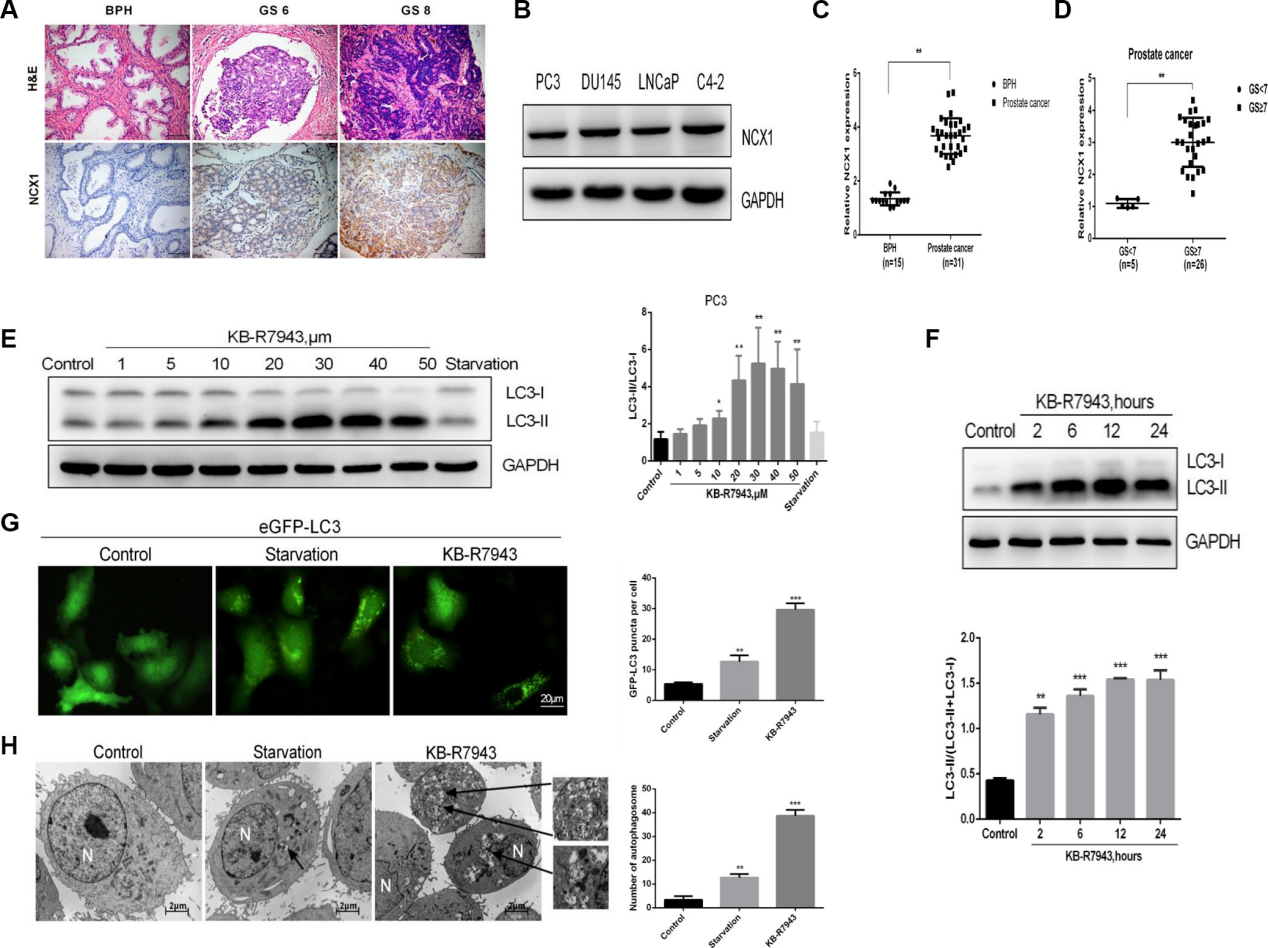
NCX1 expression was increased in the prostate cancer tissues and KB-R7943 induced autophagosome accumulation in prostate cancer cells (**A**) Results of the immunohistochemistry assay in prostate tissues. (**B**) Western blots conducted in prostate cancer cell lines. (**C**) Differences in protein levels between benign and malignant prostate tissues based on integral optical density analysis. (**D**) Differences between benign and malignant prostate tissues with different Gleason Scores. (**E** and **F**) PC3 cells were treated with the indicated concentrations of KB-R7943 for 24 h or with 30 μM KB-R7943 for the indicated periods of time, and LC3-I to LC3-II transition was analyzed using Western blotting. (**G**) PC3 cells stably expressing eGFP-LC3 (green) were treated with full or serum-starved medium or with 30 μM KB-R7943 for 24 h and examined in an immunofluorescence assay. Numbers of GFP-positive puncta per cell (*n* = 50) from three independent experiments are shown. (**H**) PC3 cells were treated with full medium, serum-starved medium, or 30 μM KB-R7943 for 24 h and examined using transmission electron microscopy analysis. Typical double-layer membrane autophagosomes (black arrows) are shown. The data are shown as means ± SD. *P < 0.05, **P < 0.01, ***P < 0.001.

The immunohistochemistry assay revealed that NCX1 expression was higher in prostate cancer tissues than in benign prostatic hyperplasia tissues (Figure 1A), and NCX1 expression increased as prostate cancer grade increased. Furthermore, western blots showed that NCX1 was expressed in all four prostate cell lines examined (PC3, LNCaP, DU145, and C4-2) (Figure 1B). Autophagy plays an important role in the regulation of prostate cancer cell survival. KB-R7943 may affect autophagy and in turn affect prostate cancer cell growth, cell cycle progression, and migration; the endogenous LC3 protein was used as a marker of autophagosome accumulation. In human prostate cancer PC3 and LNCaP cells, KB-R7943 increased LC3-II levels in a dose- (30 μM, Figure 1E) and time-dependent manner (12 h after treatment, Figure 1F). In addition, the number of eGFP-LC3 puncta increased in PC3 cells stably expressing eGFP-LC3 that were treated with KB-R7943 (Figure 1G). Furthermore, transmission electron microscopy (TEM) revealed that there were more autophagosomes in PC3 cells treated with KB-R7943 than in control cells (Figure 1H).

### KB-R7943 inhibits growth, cell cycle progression, and migration and induces apoptosis in prostate cancer cells

**Figure 2:**
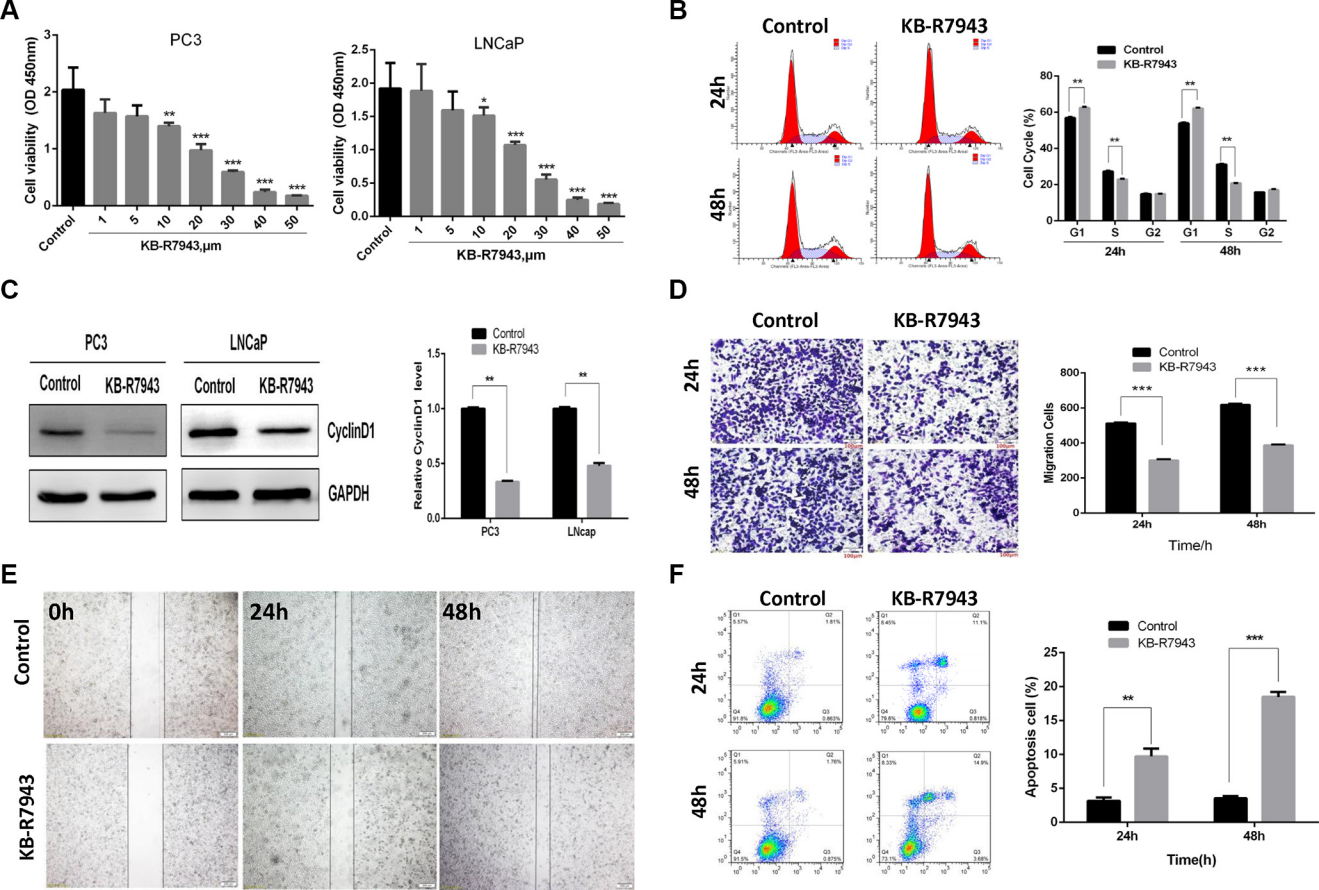
KB-R7943 inhibited prostate cancer cell growth, cell cycle progression, and migration and induced apoptosis (**A**) Both PC3 and LNCaP prostate cancer cell viability was inhibited by KB-R7943 in a CCK-8 assay. (**B**) Treatment with 30 μM KB-R7943 for 24 and 48 h inhibited cell cycle progression in a flow cytometry assay. (**C**) Immunoblotting for CyclinD1 in PC3 and LNCaP cells treated with KB-R7943 for 24 h. Densitometric analysis was used to quantify CyclinD1 normalized to GAPDH. (**D** and **E**) Treatment with 30 μM KB-R7943 for 24 and 48 h inhibited cell migration. Cell migration was examined using wound closure and transwell assays. (**F**) KB-R7943 induced apoptosis. PC3 cells were treated with 30 μM KB-R7943 for 24 and 48 h, followed by a flow cytometry assay. The data are shown as the means ± SD. *P < 0.05, **P < 0.01, ***P < 0.001.

As KB-R7943 has been reported to inhibit reverse-mode NCX1 activity in other cell models, we first confirmed the effect of KB-R7943 on PC3 and LNCaP cell proliferation by treating them with different concentrations (0, 1, 5, 10, 20, 30, 40, and 50 μM) for 24 h. The CCK-8 assay showed that KB-R7943 dose-dependently decreased viability in PC3 and LNCaP cells at concentrations of 10 μM or higher (Figure 2A). In addition, we used flow cytometry to detect changes in cell cycle distribution in PC3 prostate cancer cells induced by 30 μM KB-R7943. Compared to the normal control group, the number of PC3 cells in the G1 phase increased, while the S phase population was reduced after 24 h or 48 h of treatment (Figure 2B). Western blots revealed that the expression of CyclinD1, an important cell cycle marker, was also reduced in PC3 and LNCaP cells after KB-R7943 treatment (Figure 2C). PC3 cell migration was also suppressed after incubation with KB-R7943 (30 μM) for 24 h or 48 h in the wound healing (Figure 2D) and transwell (Figure 2E) assays. Furthermore, treatment with 30 μM KB-R7943 for 24 or 48 h induced apoptosis in PC3 cells (Figure 2F).

### KB-R7943 blocked autophagic flux

**Figure 3:**
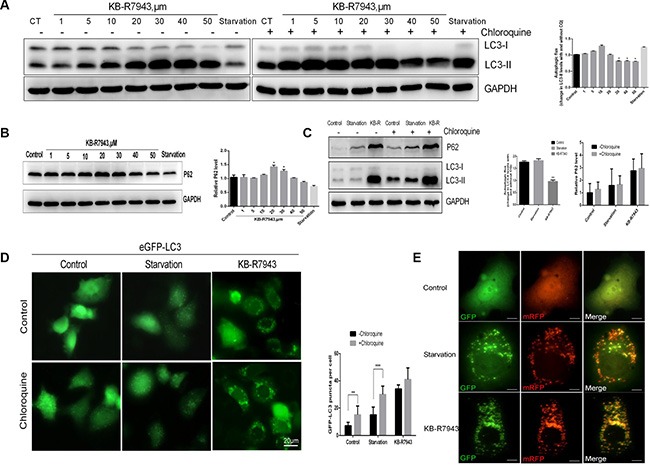
KB-R7943 blocked autophagic flux (**A**) Chloroquine treatment did not affect the KB-R7943-induced increase in LC3-II levels. PC3 cells were treated with full or serum-starved media or with 30 μM KB-R7943 for 24 h in the absence or presence of 50 μM chloroquine for the last hour. Densitometric quantitation shows autophagic flux represented by a change in chloroquine-induced LC3-II levels. (**B**) KB-R7943 increased p62 accumulation. PC3 cells were treated with full or serum-starved media or with 30 μM KB-R7943 for 24 h. Densitometric quantitation of p62 normalized to GAPDH is shown. (**C**) PC3 cells were treated with full or serum-starved media or with 30 μm KB-R7943 for 24 in the absence or presence of 50 μM chloroquine for last hour. Densitometric quantitation of autophagic flux and P62 levels normalized to GAPDH is shown. (**D**) eGFP-LC3-PC3 cells were treated with full or serum-starved media or with 30 μM KB-R7943 for 24 h in absence or presence of 50 μM KB-R7943 for the last hour. Numbers of GFP-puncta per cell (*n* = 50) are shown as means ± SD. (**E**) PC3 cells were transfected with mRFP-GFP-LC3 adenovirus and were exposed to full or serum-starved media or to 30 μM KB-R7943 as indicated. The colocalization of GFP and mRFP-LC3 puncta was examined in an immunofluorescence assay; scale bars: 10 μm. The data are shown as means ± S D, *n* = 3. *P < 0.05, **P < 0.01, ***P < 0.001.

The accumulation of autophagic vacuoles can be indicative of two different processes: increasing autophagosome formation or a reduction in autophagosome maturation/degradation. In order to investigate the mechanism of KB-R7943-induced autophagy, chloroquine (CQ), a lysosomotropic agent that blocks the fusion of autophagosomes with lysosomes and inhibits late-stage autophagy, was used to investigate autophagic flux in PC3 cells treated with KB-R7943. LC3-II protein levels gradually increased in PC3 cells after treatment with different concentrations of KB-R7943 (Figure 3A). However, this increase did not occur when cells were also treated with CQ (50 μM for 2 h) (Figure 3A). These results suggest that KB-R7943 inhibits autophagic flux at concentrations of more than 30 μM. Additionally, CQ increased eGFP-LC3 puncta accumulation in control and serum-starved PC3-eGFP-LC3 cells, but not in cells treated with KB-R7943 (Figure 3B). Protein levels of P62, another autophagy marker associated with the degradation of autophagosomes, were also examined. P62 levels changed in KB-R7943-treated PC3 cells in a dose-dependent manner, reaching a peak after treatment with 20–30 μM KB-R7943 (Figure 3C). Taken together, these results suggest that combined treatment with CQ and KB-R7943 (30 μM) decreased P62 and increased LC3-II protein levels in PC3 cells (Figure 3D).

### KB-R7943 induces autophagosome accumulation by downregulating the PI3K/AKT/m-TOR pathway and upregulating the JNK pathway

**Figure 4:**
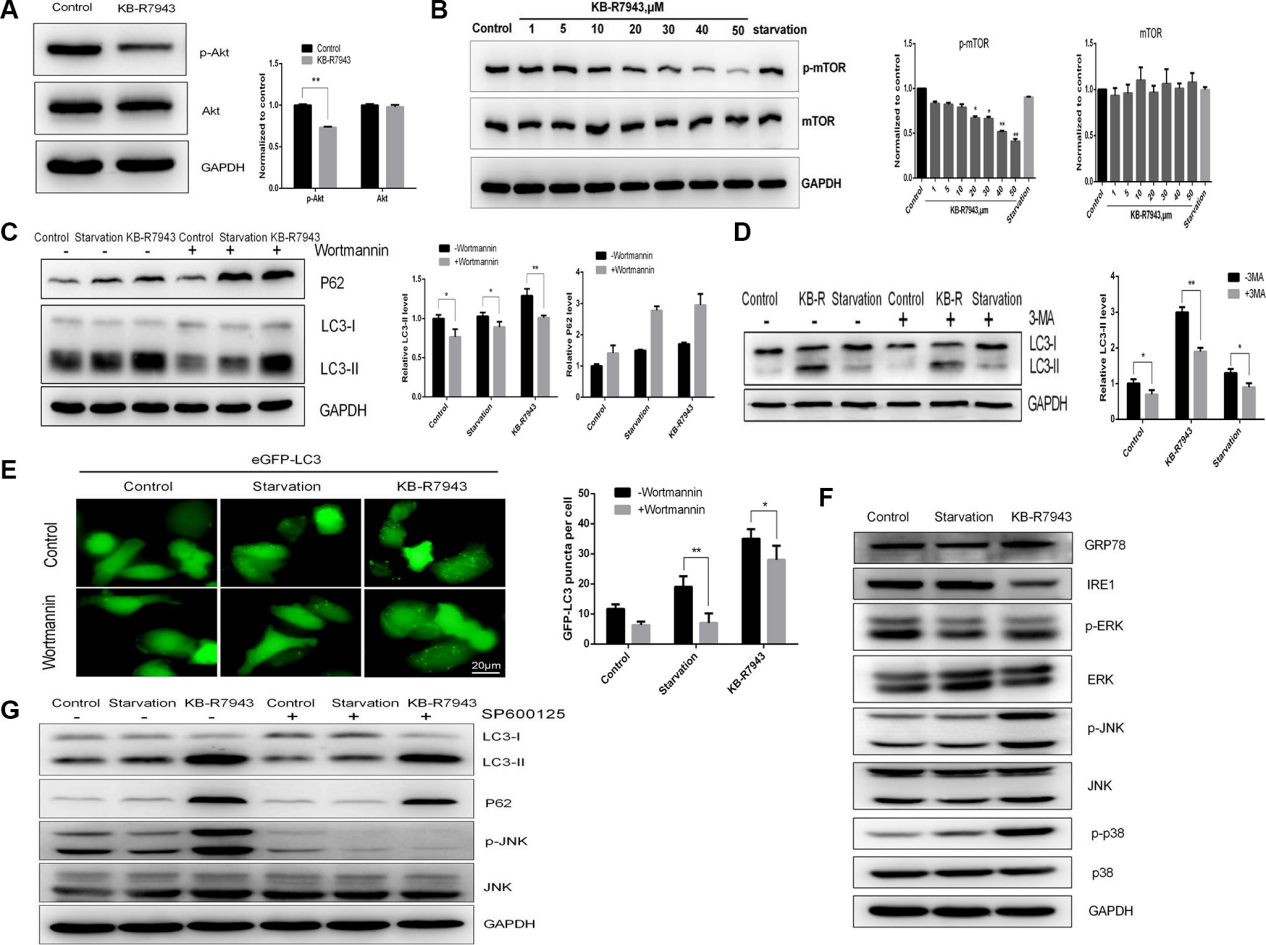
KB-R7943 induced autophagosome accumulation by downregulating the PI3K/AKT/m-TOR pathway and upregulating the JNK pathway (**A**) KB-R7943 inhibited Akt phosphorylation. PC3 cells were treated with full medium or 30 μM KB-R7943 for 24 h. Densitometric quantitation of p-Akt and Akt normalized to GAPDH is shown. (**B**) KB-R7943 inhibited mTOR phosphorylation. PC3 cells were treated with full or serum-starved media or the indicated concentrations of KB-R7943 for 24 h. Densitometric quantitation of p-mTOR and mTOR normalized to GAPDH is shown. (**C** and **D**) Wortmannin and 3-MA reduced KB-R7943-induced LC3-II levels. PC3 cells were pretreated with Wortmannin (100 nM) or 3-MA (5 mM) for 2 h followed by treatment with full or serum-starved media or 30 μM KB-R7943 for 24 h in the presence of Wortmannin (100 nM) or 3-MA (5 mM). Densitometric quantitation of LC3-II and P62 normalized to GAPDH is shown. (**E**) eGFP-LC3-PC3 cells were pretreated with Wortmannin (100 nM) for 2 h followed by treatment with full or serum-starved media or with 30 μM KB-R7943 for 24 h in the presence of Wortmannin (100 nM). (**F**) Western blot analysis of p-ERK, ERK, p-JNK, JNK, p-p38, p38, GRP-78, IRE1, and GAPDH levels in cells treated with full or serum-starved media or with 30 μM KB-R7943 for 24 h. (**G**) Western blot analysis of p-JNK, JNK, LC3-II, p62, and GAPDH levels in cells treated with full or serum-starved media or with 30 μM KB-R7943 in the presence or absence of 10 μM SP600125 for 24 h. The data are shown as means ± SD, *n* = 3. *P < 0.05, **P < 0.01, ***P < 0.001.

AKT phosphorylation plays an important role in tumor survival and development, and the Akt/mTOR signaling pathway is a classical autophagy pathway. AKT phosphorylation levels were reduced in PC3 cells treated with 30 μM KB-R7943 (Figure 4A). mTOR phosphorylation levels also decreased dose-dependently in PC3 cells after treatment with KB-R7943 (Figure 4B). To further investigate the mechanism of KB-R7943-induced autophagosome accumulation in prostate cancer cells, we treated PC3 cells with Wortmannin, which inhibits phopshatidylinositol 3-kinase and the initial stages of autophagy. LC3-II levels decreased in PC3 cells treated with KB-R7943 (30 μM) plus Wortmannin compared to PC3 cells treated with KB-R7943 alone, but P62 protein levels increased in cells receiving combined treatment (Figure 4C). 24 h of pretreatment with 5 mM 3-MA, another Vps34 inhibitor, inhibited LC3-II levels at baseline and after treatment with full, serum-starved, or KB-R7943 (30 μM) containing medium in PC3 cells (Figure 4D). Meanwhile, in PC3-eGFP-LC3 cells, the number of LC3-puncta decreased after treatment with KB-R7943 (30 μM) plus Wortmannin (Figure 4E). JNK is a member of the MAPK signaling pathway family, which regulates a variety of biological processes, such as survival, proliferation, and differentiation. Treatment with 30 μM KB-R7943 increased JNK and p38 phosphorylation in PC3 cells. However, the ERK phosphorylation levels did not change (Figure 4F). The JNK inhibitor SP600125 (10 μM) was used to confirm the above observation. Interestingly, pretreatment of PC3 cells with SP600125 reduced LC3-II levels after KB-R7943 treatment (Figure 4G). These results suggest that JNK is involved in KB-R7943-induced autophagosome accumulation.

### Combination therapy and inhibition of autophagy reduces prostate cancer cell growth

**Figure 5:**
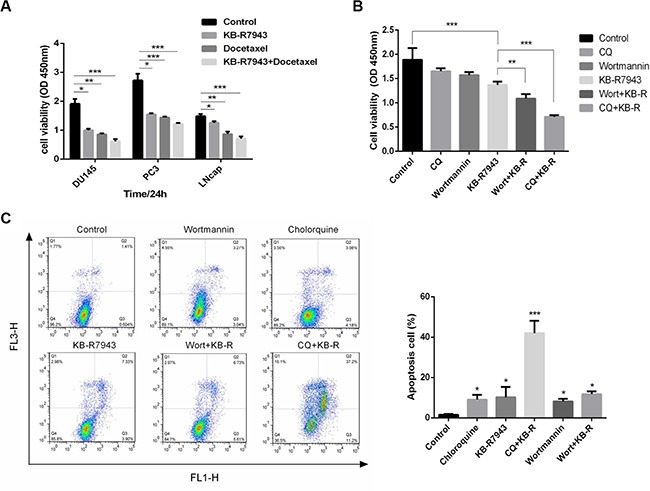
The effects of combination therapy and inhibition of autophagy in prostate cancer cells (**A**) DU145, PC3, and LNCaP cells were treated with docetaxel (5 nM), KB-R7943 (30 μM), or a combination of docetaxel and KB-R7943 for 24 h. Cell viability was monitored using the CCK-8 assay. (**B**) PC3 cells were treated with KB-R7943 (30 μM) in the presence or absence of chloroquine (50 μM) or Wortmannin (100 nM) for 24 h. Cell viability was measured using the CCK-8 assay. (**C**) Cells were treated with KB-R7943 alone or in combination with CQ or 3-MA before staining with Annexin V (AV) and propidium iodide (PI), followed by a flow cytometry assay. The data are shown as means ± SD, *n* = 3. *P < 0.05, **P < 0.01, ***P < 0.001.

Combined treatment with KB-R7943 and docetaxel (5 nM), a chemotherapy drug used to treat prostate cancer, reduced cell viability (Figure 5A). Autophagy may act as a stress-activated pro-survival mechanism in cancer cells. Joint application of KB-R7943 and an autophagy inhibitor inhibited growth and increased the apoptosis rate (Figure 5B and 5C).

### KB-R7943 inhibits PC3 xenograft tumor growth *in vivo*

**Figure 6:**
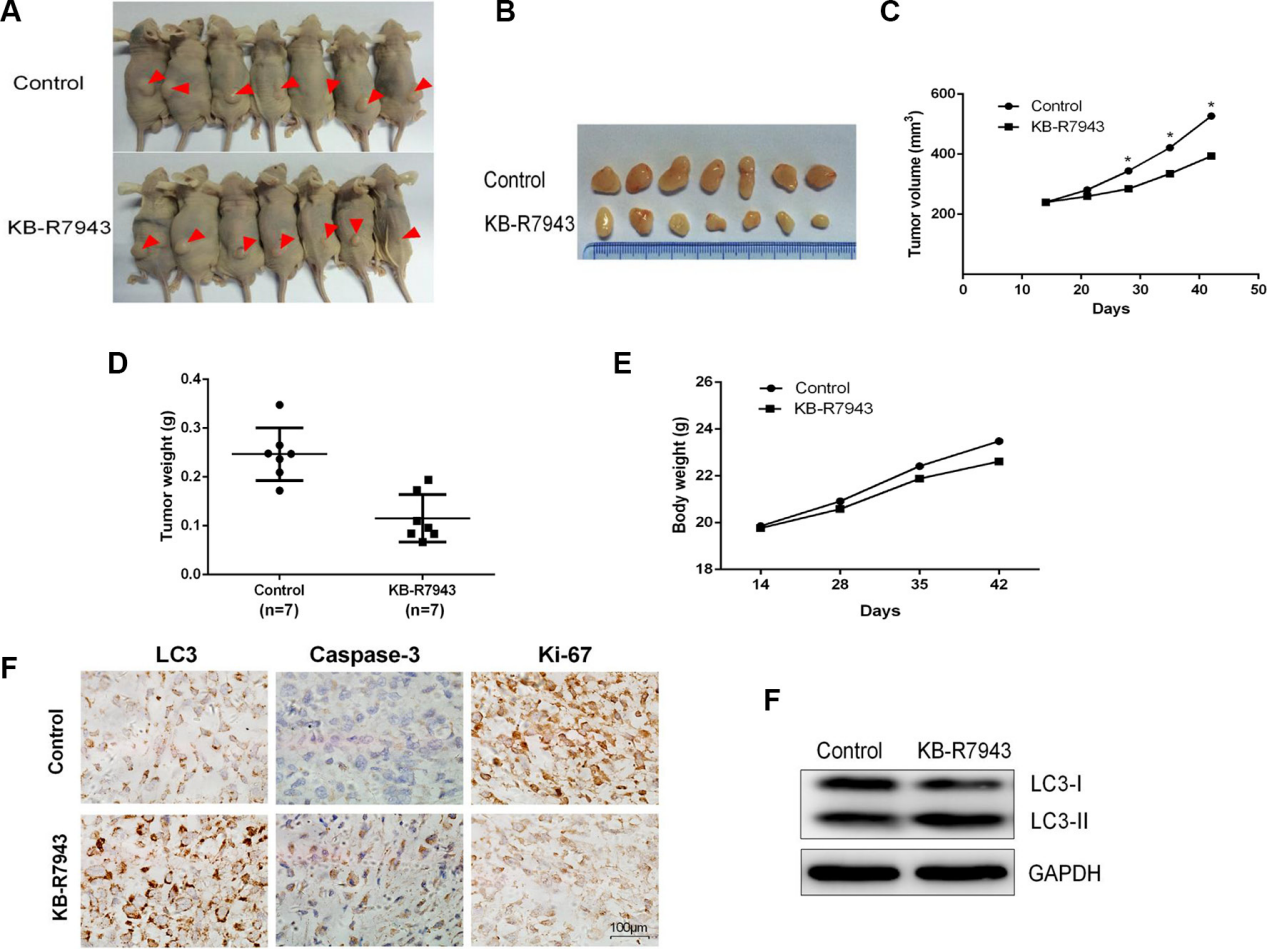
KB-R7943 inhibited PC3 xenograft tumor growth *in vivo* 2 × 10^6^ PC3 cells were injected subcutaneously into male nude mice. Mice with tumors of similar sizes were randomly separated into two groups (7 mice/group). The control group received daily IP injections of PBS, and the treatment group received daily injections of KB-R7943 at 10 mg/kg. (**A**, **B**, **C**, **D**, **E**) Tumor volumes and weights and body weights were measured once a week. One-way ANOVAs and *t*-tests for pairwise comparison were used to analyze data. (**F** and **G**) LC3-II, cleaved caspase-3, and Ki-67 levels in tumors were examined using immunohistochemistry, and LC3-II levels were also examined using western blot.

The above observations suggest that KB-R7943 inhibits prostate cancer cell growth *in vitro*. To confirm that KB-R7943 had the same effect *in vivo*, we subcutaneously injected PC3 cells into male nude mice. KB-R7943 inhibited the growth of tumors resulting from PC3 cell xenografts (Figure 6A). More importantly, Ki-67 levels decreased and LC3 and caspase 3 levels increased (Figure 6B), indicating that KB-R7943 inhibited tumor growth by inducing autophagosome accumulation and apoptosis.

## DISCUSSION

The role of NCX1 in prostate cancer has not been widely studied. Here, we found that NCX1 was aberrantly expressed in prostate cancer cells and tumor tissues (Figure 1A and 1B), especially in higher-grade PCa tissues. In order to investigate the role of NCX1 in prostate cancer, we treated cells with the drug KB-R7943, which inhibits reverse-mode NCX1 activity. As shown in the schematic in Figure 2, KB-R7943 not only promoted apoptosis both in PC3 and LNCaP cells, but also induced cell cycle arrest, especially in the S phase. KB-R7943 also inhibited the migration of PC3 cells in wound healing and transwell assays *(p <* 0.05). Thus, NCX1 plays a vital role in prostate cancer growth and survival.

Autophagy is closely related to cancer growth and may be a target for cancer therapy. Here, we demonstrated for the first time that NCX1 contributes to the development of prostate cancer. First, to examine the effect of NCX1 inactivation on autophagy, we treated prostate cancer cells with KB-R7943. Our results indicate that KB-R7943 blocks autophagic flux and inhibits the degradation of autophagosomes. The accumulation of LC3-II has been used to measure autophagic flux. Concentration-dependent decreases in PC3 and LNCaP cell viability were observed after treatment with KB-R7943 at concentrations greater than 10 μM. Therefore, we examined changes in LC3-II and p62 after treatment with Chloroquine, an antibiotic used to block autophagy, in combination with KB-R7943. As expected, compared with the control groups, LC3-II and p62 levels measured using transmission electron microscopy were decreased by the combined treatment. In conclusion, the KB-R7943-induced inhibition of autophagic flux reduces growth and survival in prostate cancer.

Some studies suggest that increased intracellular Ca^2+^ can promote autophagy [24–26]. KB-R7943, as an inhibitor of reverse-mode NCX1 activity, inhibits the influx of Ca^2+^ into cells. A previous study demonstrated that NCX1 participates in neuronal differentiation through ionic regulation and Akt phosphorylation [27]. Here, KB-R7943 induced autophagosome accumulation by inhibiting mTOR kinase in a dose-dependent manner in prostate cancer cells. In addition, KB-R7943 inhibited the phosphorylation of AKT. These results indicate that KB-R7943 stimulated autophagosome accumulation mainly by inhibiting the AKT/mTOR signaling pathway. Changes in autophagic flux, which refers to the complete process of exchange of material to lysosomes and subsequent breakdown and recycling, can be indicative of either an increase or decrease in autophagy. It is important to examine the role of autophagy in cancer progression and the ways in which treatment affects this process [28]. Our results confirm that KB-R7943 can block autophagic flux in PC3 cells, suggesting that KB-R7943 may affect lysosomal pH and inhibit the degradation of autophagosomes. Cation and anion homeostasis directly affects pH and membrane potential in lysosomes [29]. Recent reports suggest that NAADP-induced Ca^2+^ release from lysosomes via TPC2 is accompanied by increased lysosomal pH [30]. In our study, treatment with KB-R7943 may have increased lysosomal pH in PC3 cells by affecting interactions between Ca^2+^/Na^+^ exchangers and Na^+^/H^+^ exchangers; H^+^ concentrations in the lysosomes may have increased when Ca^2+^ concentrations decreased. Therefore, our findings suggest that KB-R7943 may inhibit autophagic flux by blocking autophagosome-lysosome fusion.

The mechanisms by which KB-R7943 inhibits proliferation and promotes apoptosis in prostate cancer cells are still unclear. One study suggested that autophagy may be upregulated under conditions of endoplasmic reticulum (ER) stress, eventually inducing cell death [31]. Another study showing that IRE1 activates the TRAF2/JNK pathway in neuroblastoma cells confirmed the connection between ER stress and autophagy [32]. Similarly, our findings suggest that KB-R7943 induced ER stress by activating p38 and JNK signaling in addition to increasing apoptotic cell death. We further confirmed these results using SP600125, an inhibitor of the JNK pathway, which reduced LC3-II and P62 levels in KB-R7943-treated prostate cancer cells. The JNK signaling pathway has been reported to regulate apoptosis under ER stress conditions [33]. Thus, JNK activation might induce apoptosis in response ER stress, ultimately resulting in cell death. Interestingly, KB-R7943 treatment increased GRP78 levels and decreased IRE1 levels. Another recent report showed that IRE1 prevents ER membrane permeabilization and cell death under pathological conditions [34]. KB-R7943 may therefore cause cancer cell death by permeabilizing the ER membrane. In addition, apoptosis is closely related to ER stress during chemotherapy [35]. Here, the autophagy inhibitor CQ enhanced the apoptosis-inducing effect of KB-R7943 treatment in prostate cancer cells. The combination of these two approaches may increase the efficacy of anti-tumor therapies. In addition, KB-R7943 also increased the anti-cancer activity of docetaxel, suggesting a potential role for KB-R7943 as an adjuvant to anti-cancer chemotherapy. Therefore, these combination therapies may provide new treatment strategies for prostate cancer.

**Figure 7:**
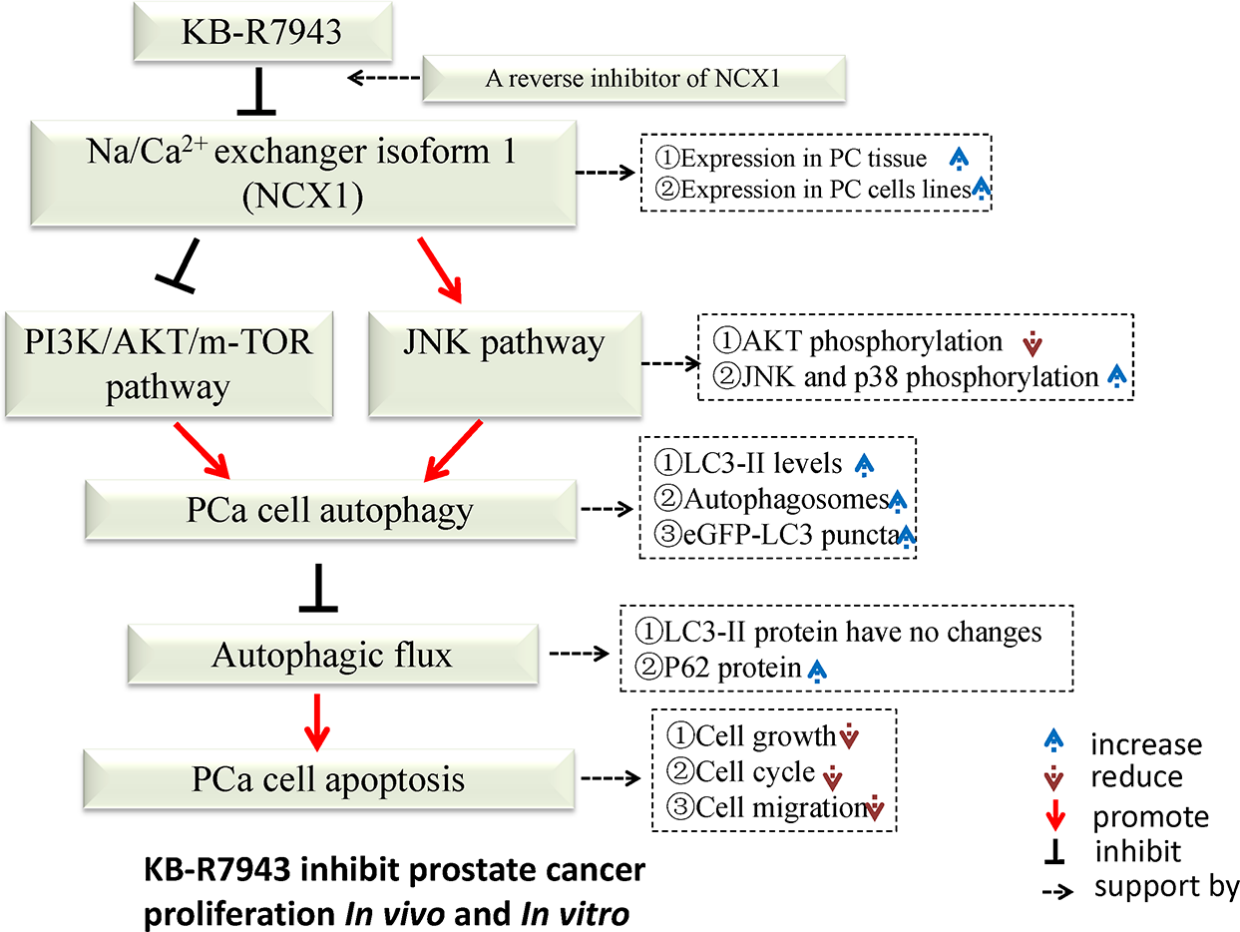
Schematic description of the relationship between autophagy and apoptosis in PC3 cells following KB-R7943 treatment

In summary (Figure 7), we demonstrated that NCX1 is highly expressed in prostate cancer and that the reverse-mode NCX1 activity inhibitor KB-R7943 can inhibit cell cycle progression, migration, and proliferation both *in vivo* and *in vitro*. Additionally, KB-R7943 can induce autophagosome accumulation and apoptosis in prostate cancer cells. KB-R7943 also activates the JNK signaling pathway and blocks autophagic flux, which promotes cell death in prostate cancer. Finally, KB-R7943-induced inhibition of autophagic flux increases apoptosis in prostate cancer. Our findings suggest that KB-R7943 may be a potential treatment for prostate cancer.

## MATERIALS AND METHODS

### Antibodies and reagents

Rabbit anti-SQSTM1/P62 (D5E2), rabbit anti-LC3A/B (D3U4C), rabbit anti-mTOR (7C10), rabbit anti-p-mTOR (Ser2448), rabbit anti-p-SAPK/JNK (Thr183/Thr185), rabbit anti-SAPK/JNK (56G8), rabbit anti-cyclinD1 (96G2), rabbit anti-p-ERK (Thr202/Thr204), rabbit anti-ERK (137F5), rabbit anti-p-P38 (Thr180/Tyr12), rabbit anti-P38 (D13E1), rabbit anti-p-AKT (Ser473, D9E), and rabbit anti-AKT (C67E7) were from Cell Signaling (Danvers, MA, USA). Rabbit anti-GRP78 (ab21685), rabbit anti-IRE1 (ab37073), rabbit anti-NCX1 (ab151608), and rabbit anti-Ki67 (ab15580) were from Abcam (Cambridge, MA, USA). Mouse anti-GAPDH was from Beyotime Biotechnology (Nanjing, China). NCX1 inhibitor KB-R7943 (Sigma, St Louis, MO, USA, K4144-25MG) was dissolved in DMSO at 10 mM. Autophagy inhibitor 3-methyladenine (Sigma, M9281-100MG) was dissolved in medical injectable water at 50 mM. Chloroquine (Sigma, C6628-25G) was dissolved in medical injectable water at 10 mM. Wortmannin (Beyotime Biotechnology, Nanjing, China) and SP600125 (Sigma, S5567-10MG) were dissolved in DMSO at 10 mM.

### Cell culture and transfection

Human prostate cancer cell lines PC3, LNCaP, DU145, and C4-2 were obtained from the Cell Collection Center of Peking Union Medical School in China. PC3 cells were cultured in F12k medium (Gibco-Life Technologies, Grand Island, NY, USA) and supplemented with 10% fetal bovine serum (Gibco-Life Technologies) and 1% antibiotics (Beyotime Biotechnology, Nanjing, China). LNCaP, DU145, and C4-2 cells were cultured in RPMI-1640 medium (Gibco-Life Technologies).

PC3 cells stably expressing eGFP-LC3 were generated by transfection with eGFP-LC3 plasmid (Addgene, USA) using X-treme GENE HP transfection reagent (Roche, USA), and G418 (500 μg/mL) was added to select positive cells. PC3 cells were seeded on 24-well plates and infected with GFP-mRFP-LC3 adenovirus for 24 h; subsequently, cells were either treated with full medium, KB-R7943 (30 μM), or serum-starved for 24 h. Images were visualized using an Olympus fluorescence microscope system.

### Cell viability assay

Cells were seeded at 5000 cells/well on 96-well plates in normal medium and cultured for 24 h. Cells at approximately 70% confluence were then treated either with full medium or with the indicated concentrations of KB-R7943 in serum-starved media for 24 h. In addition, cells were treated with KB-R7943, CQ, Wortmannin, Docetaxel, or combinations of these drugs. After treatment, cell viability assays were performed using the Cell Counting Kit-8 (Dojindo Laboratories, Kumamoto, Japan). At the indicated time points, cells were treated with CCK-8 at 10 uL/well at 37°C for 2 h, and the numbers of cells per well were determined by measuring absorbance at 450 nm.

### Western blotting

Total protein was extracted from lysates as previously described, and total protein concentration was measured using the RCDC method (BIO-RAD, USA). Samples were separated by SDS-PAGE, transferred to PVDF membranes (Millipore, USA), and incubated with the following primary antibodies at 4°C overnight: LC3A/B, p-mTOR, mTOR, SQTM1/P62, CyclinD1, p-JNK, JNK, p-ERK, ERK, p-p38, P38 (1:1000, Cell Signaling, USA); GRP78, IRE1, NCX1 (1:1000, Abcam, USA); GAPDH (1:500, Beyotime Biotechnology, Shanghai, China). Membranes were then incubated with goat anti-mouse and goat anti-rabbit secondary antibodies (1:5000, Zhongshan Company, Beijing, China). Proteins were visualized using ECL (Millipore, Bradford, MA, USA) and detected using the Image Quant LAS-4000 (Fujifilm, Tokyo, Japan) BioImaging System.

### Flow cytometry

PC3 cells were treated with full medium alone or with 30 μM KB-R7943 for 24 h or 48 h. Cell cycle arrest was determined by measuring the incorporation of PI (Beyotime, Nanjing, China) into permeabilized cells according to the manufacturer's protocol. PC3 cells were treated with full medium (Control) alone, 30 μM KB-R7943, CQ, Wortmannin, or a combination for 24 h. Cell apoptotic rate was determined using an Annexin V-FITC kit (Beyotime, Nanjing, China) following the manufacturer's instructions. The cells were analyzed using a CoulterEpics XL flow cytometer (Beckman Coulter, Miami, FL, USA).

### Monolayer wound healing and matrigel invasion assays

PC3 cells were seeded in a 24-well plate, allowed to reach 70% confluence, and scratched with a 10 μL plastic pipette tip to create a wound. Wounds were then treated with KB-R7943 (0 or 30 μM). Cell migration (wound healing) was photographed at 0, 24, and 48 hours. A 24-well Transwell chamber with 8 μm pores was used for the cell matrigel invasion assay (Millipore, USA). Cell were seeded into the upper chambers with serum-free medium or KB-R7943 (30 μM) at a density of 1 × 10^5^ cells, and culture medium containing 10% fetal bovine serum was added to the lower chamber. After incubation for 24 or 48 h, cells were washed with PBS, fixed with 4% paraformaldehyde for 20 min, and stained with 0.1% crystal violet 15 min. The numbers of migrated/invading cells were counted in three random fields using a light microscope. All experiments were done in triplicate.

### Immunohistochemistry

This study was conducted with the approval of the ethics committee of Third Military Medical University. Samples from 31 prostate cancer cases and 15 benign prostate hyperplasia cases were obtained from the Second Affiliated Hospital of Third Military Medical University between 2012 and 2015. None of the patients had received radiation therapy or chemotherapy before surgery. Histological diagnosis, including the evaluation of hematoxylin and eosin staining, was performed according to World Health Organization guidelines. Paraffin-embedded tumor tissue sections (4 μm) were deparaffinized with xylene and rehydrated in a graded ethanol series. Sections were stained with anti-LC3A/B, anti-caspase3, anti-NCX1, anti-Ki67, and secondary anti-mouse IgG using the streptavidin-biotin peroxidase conjugated (SP) two-step method and a standard SP kit (Zhongshan Biotech, Beijing, China) according to the manufacturer's protocol. Two independent investigators examined all tumor slides randomly for classification.

### Transmission electron microscopy

PC3 cells were seeded at 1 × 10^6^ cells/well in 6-well plates and treated with designated concentrations of KB-R7943 for 24 h. After treatment, cells were washed and fixed for 30 min in 2.5% glutaraldehyde. The samples were then treated with 1.5% osmium tetroxide, dehydrated with acetone, and embedded in Durcupan resin. The sections were subsequently examined under a transmission electron microscope (JEOL JEM-1200EX, Japan) at 60 kV.

### Xenograft tumor assay

Animal experimental protocols were approved by Third Military Medical University Institutional Animal Care and Use Committee. Male nude mice (4–5 weeks old) were purchased from Fu Kang Biological Technology (Beijing, China), fed a standard animal diet, and given water. PC3 cells (1 × 10^6^) were inoculated into the backs of the mice on the right side. 14 days after tumor inoculation, the mice were randomly divided into two groups (7 mice per group) and intraperitoneally injected daily with buffer only (control) or 10 mg/kg KB-R7943. Tumor sizes and body weights were measured once a week. Tumor diameters were measured and the tumor volume (mm^3^) was calculated as follows: volume = (shortest diameter)^2^ × (longest diameter)/2. The animals were killed after 30 days of injections, and tumors were weighed and used for western blot or paraffin embedding. The remaining tumor was frozen in liquid nitrogen. Immunohistochemical assays were performed as previously described. Western blots were performed using tumor lysates with LC3 antibody.

### Statistical analysis

Error bars and *P* values were calculated using three different assays for each experiment. *P* values for comparisons between groups were obtained using unpaired *t*-tests or one-way analyses of variance (ANOVA) using GraphPad Prism 6 software (GraphPad, San Diego, CA, USA). *P* < 0.05 was considered statistically significant.
